# Nevirapine-associated liver toxicity and hypersensitivity reactions in a cohort of HIV-1-infected patients,clinical analysis

**DOI:** 10.1186/1742-4690-9-S1-P10

**Published:** 2012-05-25

**Authors:** Sylvie Jonckheere, JC Yombi, Leila Belkhir, Anne Vincent, Bernard Vandercam

**Affiliations:** 1 Medecine Interne Infectiologie at Centre Refrence St Luc Ucl, Bruxelles, Belgium

## Introduction

Antiretroviral drug-related liver injury is a common cause of morbidity and treatment discontinuation in HIV-infected patients. Nevirapine is incriminated as one of the liver toxicity inducer especially in patients with high CD4-cells count. The purpose of our study was to analyze the role of CD4 cell count at treatment initiation and that of several co-factors (Hepatitis C or Hepatitis B virus co-infection, concurrent use of protease inhibitors) on the incidence of liver toxicity and hypersensivity reactions induced by Nevirapine in our HIV1-infected patients.

## Material and method

We analyzed retrospectively a cohort of 930 HIV-1 infected patients. Patients, who were started on NVP between 1998 and 2003, regardless of CD4-cells count, were included. We assigned patients to two groups: (A) group with high CD4-cells counts (women with CD4 cells ≥ 250 cells/mm³ and men with ≥ 400 cells/mm³), and (B) group with low CD4-cell counts. Liver toxicity is considered severe when at least a grade 3 toxicity is observed (WHO classification).

## Results

In total 108 patients were included. Eight (7.40%) and 15 (13.9%) patients interrupted treatment because of severe liver toxicity and hypersensitivity reactions respectively. There was no overlap between these two groups, and hypersensitivity reactions tended to occur sooner (22 vs 45 days respectively). HCV and HBV co-infection rates were 7.4% and 8.33%. Severe liver toxicity was seen in 15.74% of patients. Comparing group A and B, rates of severe liver toxicity were 15.68% and 17.30% respectively. There was no significant difference. In a multiple linear regression model, we found viral hepatitis C co-infection to be the only independent risk factor in the occurrence of liver toxicity (p<0.006).

## Conclusion

In our study the rate of severe liver toxicity due to NVP was high. HCV co-infection was an independent risk factor for liver toxicity, contrary to CD4 cell counts at treatment initiation. These findings are in keeping with recent published data. A careful analysis of the literature shows that hypersensitivity reactions due to NVP are strongly correlated with high CD4 cell counts. The limitation of our study is the low number of patients included.

